# ggbulksurv: An R package for easy Drosophila and C. elegans survival analysis

**DOI:** 10.17912/micropub.biology.001060

**Published:** 2024-05-03

**Authors:** Qian Hui Tan, Nathan Harmston, Nicholas S Tolwinski

**Affiliations:** 1 Division of Science, Yale-NUS College, Singapore, Singapore, Singapore; 2 Program in Cancer and Stem Cell Biology, Duke-NUS Medical School, Singapore, SG.01, Singapore

## Abstract

Lifespan studies on fast-aging model organisms like *C.elegans* and *D.melanogaster* are conducted with multiple organisms per vial. Lifespan data results in a “one row, multiple individuals” format, which is incompatible with R packages that require a “one row, one individual” format. We present *ggbulksurv* , an R package for user-friendly survival analysis and highlight three key features. (1) *pivot_prism* converts data for PRISM, allowing biologists to plot survival curves without manually expanding each observation. (2) *run_bulksurv()* takes in a “one row, multiple individuals” table and plots a customizable survival curve. (3) Advanced users who require custom survival objects can specify a custom formula, facilitating complex survival analysis. We provide a time saving solution for lifespan data analysis.

**Figure 1. A graphical overview of ggbulksurv:**
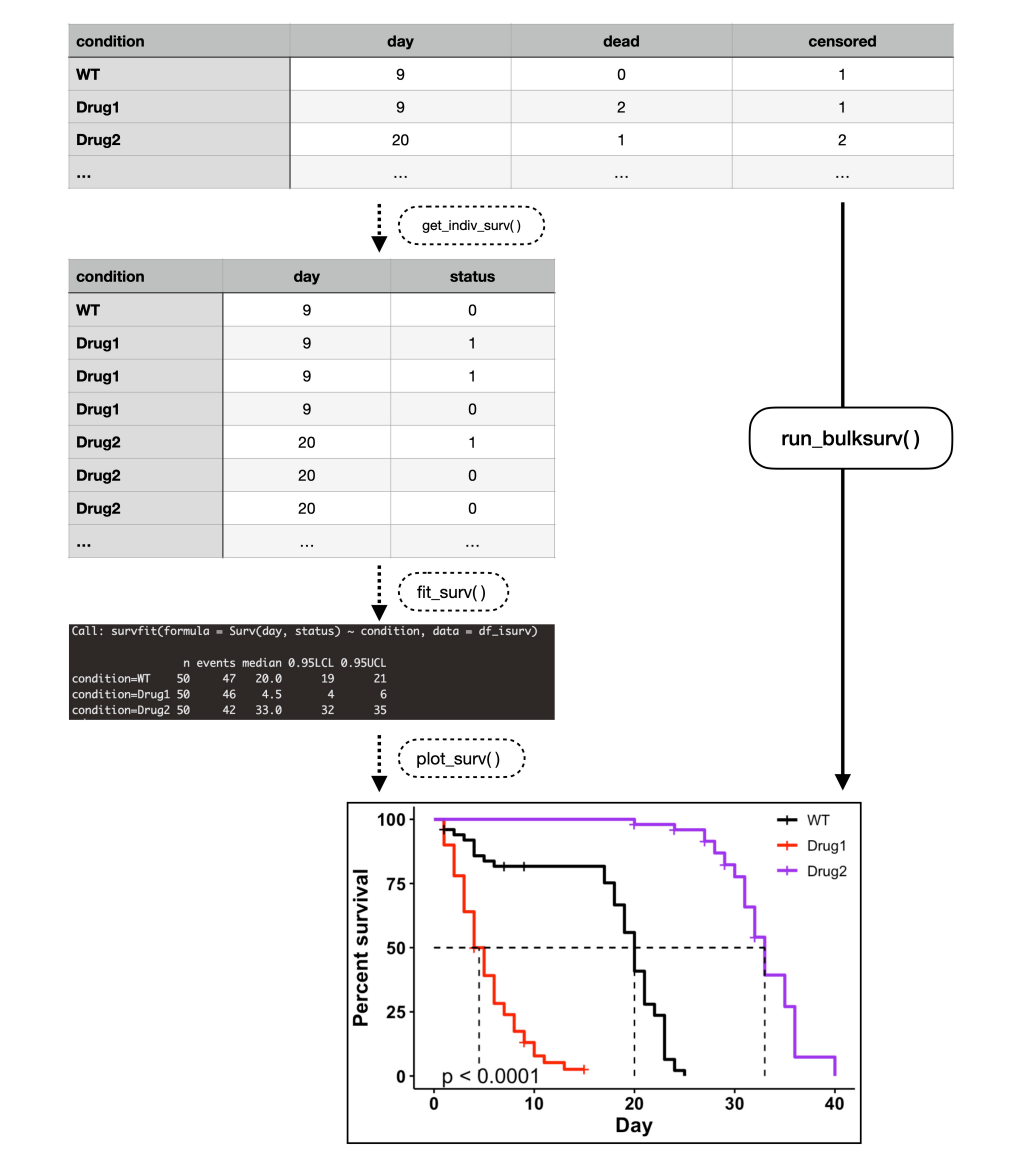
*(1) get_indiv_surv()* transforms a “one row, multiple individuals” table into a “one row, one individual” table. (2) *fit_surv()* fits a survival object using the default call `Surv(day, status) ~ condition`. (3) *plot_surv()* plots the survival curve. (4) *run_bulksurv()* is a shortcut which sequentially runs *get_indiv_surv()* , *fit_surv()* and *plot_surv()* . To specify a custom formula, use the *formula* parameter in *run_bulksurv().* Data plotted here is a fictional dataset bundled with the *ggbulksurv* package.

## Description

Human and mouse lifespan studies assign a unique ID to each individual to follow their entire lifespan. Most survival packages in R ( *survival* (Therneau, 2020) *, survminer* (Kassambara et al., 2021) *)* thus require survival data to be entered in a “one row, one individual” format, where each row corresponds to a single individual and their accompanying data (Extended Data 1a).

This data format is incompatible with lifespan studies in model organisms such as *C.elegans* and *D. melanogaster* , where multiple organisms are placed in the same vial or plate. Instead of assigning each worm or fly a unique ID, researchers count the number of dead and censored organisms each day. This produces a “one row, multiple individuals” table (Extended Data 1b, 1c), and is thus incompatible with the input format required by GraphPad PRISM v10.2.3 (Extended Data 1d) and survival packages in R.

Several tools have been developed to tackle this problem. For example, OASIS2 (Han et al., 2016) allows users to input a “one row, multiple individuals” table for survival analysis, and only requires 3 columns: day, dead, censored. OASIS2 plots are however not customizable, and statistical analysis tests are unable to accommodate complex study designs. Another alternative is WormLifespanPlot (WLSplot) (Mariner et al., 2023) , an R package for C.elegans lifespan analysis. While WLSplot allows users to input a “one row, multiple individuals” table, it requires specific parameters such as FUDR usage, worm strain information along with the full lifespan table in a pre-formatted Excel sheet, which may not be relevant for other organisms such as Drosophila (Extended Data 1b). Furthermore, WLSplot does have limitations as it does not allow for p-value corrections for multiple comparisons, and does not allow users to add additional annotations such as confidence intervals.

The *ggbulksurv* ( **gg** plot2-based **bulk** **surv** ival analysis) package addresses this problem by automating the survival analysis pipeline, enabling users to run R survival analysis with the *run_bulksurv()* wrapper function. This function pivots data, plots a publication-ready survival curve, and runs a default statistical analysis for median survival and log-rank test. For PRISM users, *ggbulksurv* also provides a *pivot_prism()* function that allows users to easily convert their “one row, multiple individuals” data to a PRISM-compatible format.

**Package overview**

The *ggbulksurv* package can be accessed at https://github.com/qhuitan/ggbulksurv . *ggbulksurv* requires a csv file as input. Similar to OASIS2, this csv file must have at least four columns: condition, day, dead, censored. Additional columns (eg. sex, genotype, treatment) can be added.

After reading the lifespan csv file into R, users can use *run_bulksurv()* to perform a default survival analysis. By default, this fits a survival object on `condition`, ignoring other columns present. Users can specify custom survival formula within *run_bulksurv()* .

The *run_bulksurv()* function returns the survival curve as a *ggplot2* object, which can be further manipulated in R. In addition, the function prints the results of three statistical tests – median survival, log-rank test and a pairwise log-rank test between all conditions. All statistical tests default to the Benjamini-Hochberg (BH) method for p-value adjustment, but other options such as Bonferroni are also available.

Besides survival curves, *run_bulksurv()* also allows users to plot a mortality plot using the parameter type = “mortality”. Other popular customization options include the addition of confidence intervals, custom colors, and median survival lines.

**Behind the scenes: How ggbulksurv works**

The default analysis pipeline consists of three stages. (1) *get_indiv_surv()* converts a “one row, multiple individuals” table to a “one row, one individual” table ( Figure 1a, 1b). (2) *fit_surv()* fits a survival object using the R *survival* package using the call `Surv(day, status) ~ condition` ( Figure 1c ). (3) *plot_surv()* generates the required lifespan plots by wrapping around the *ggsurvplot* function from the *survminer* package ( Figure 1d ). All additional inputs to *plot_surv()* are passed to *survminer::ggsurvplot()* , though some default values are set for the user.

Median survival is calculated by *fit_surv()* , which prints the median survival from the R *survival* package. The log-rank test is performed using *survival::survdiff()* , while the pairwise log-rank test is performed using *survminer::pairwise_survdiff()* . For all survival statistics, an R survival object is fit with the command `Surv(day, status) ~ condition` by default. To specify a custom study design, use the *formula* parameter. For example, *run_bulksurv(data, formula = `Surv(day, status) ~ condition + sex`)* would fit a survival object on both condition and sex.

**Converting to PRISM**

GraphPad PRISM is a user-friendly software for survival analysis, but it requires a specific input format (Extended Data 1d). Data is often entered manually, for example, if 15 individuals in the Control group died on day 10, users would manually type 15 rows of 1s in the Control column of GraphPad PRISM.

The *ggbulksurv* package provides a *pivot_prism()* function that allows users to convert a “one row, multiple individuals” input format into a PRISM-compatible format. After reading in the csv file, users can run the *pivot_prism()* function to pivot the data, then use *write.csv()* to export. The csv file can be copied and pasted into GraphPad PRISM.

**Summary and discussion**

The *ggbulksurv* package presented here provides a simple solution for lifespan data analysis from *C.elegans* and *D.melanogaster* studies. *ggbulksurv* provides users with simple functions that convert the “one row, multiple individuals” bulk survival format into a “one row, one individual” format, allowing it to be compatible with other R survival packages. In addition, *run_bulksurv()* automates lifespan analysis by plotting Kaplan-Meier survival curves and returns three important statistics – median survival, log-rank test and a pairwise log-rank test between conditions. We further provide a function that allows users to transform “one row, multiple individuals” data into a GraphPad PRISM compatible format using the *pivot_prism()* function. We hope that this package provides a time saving solution for the field.

## Extended Data

Description: Examples of plots and survival statistics generated by ggbulksurv.. Resource Type: Image. DOI: 10.22002/nrnct-79544

Description: An R package for bulk survival analysis. Resource Type: Software. DOI: 10.22002/h4tv8-at129
